# Nonrigid Medical Image Registration Based on Mesh Deformation Constraints

**DOI:** 10.1155/2013/373082

**Published:** 2013-02-03

**Authors:** XiangBo Lin, Su Ruan, TianShuang Qiu, DongMei Guo

**Affiliations:** ^1^Faculty of Electronic Information and Electrical Engineering, Dalian University of Technology, Dalian 116024, China; ^2^Laboratory of Computer Science, Information Processing and Systems, University of Rouen, 76183 Rouen, France; ^3^Department of Radiology, Second Affiliated Hospital of Dalian Medical University, Dalian 116027, China

## Abstract

Regularizing the deformation field is an important aspect in nonrigid medical image registration. By covering the template image with a triangular mesh, this paper proposes a new regularization constraint in terms of connections between mesh vertices. The connection relationship is preserved by the spring analogy method. The method is evaluated by registering cerebral magnetic resonance imaging (MRI) image data obtained from different individuals. Experimental results show that the proposed method has good deformation ability and topology-preserving ability, providing a new way to the nonrigid medical image registration.

## 1. Introduction

Deformable registration is an important tool in medical imaging with many applications, such as atlas-based image segmentation and labeling, statistical analysis of normal and pathological variations in anatomy, and the study of the growth and development of normal and abnormal anatomical structures. It has been an active research topic for many years, and plenty of achievements have been published [1, 2]. The basic task of image registration is to find a spatial transformation which maps each point of one image onto its corresponding point of another image. The problem of image registration is often considered as a minimization problem, because it looks for increasing some similarity metric between the two images to be registered by moving points with a reasonable deformation field. Common choices of image similarity metric include sum of squared differences (SSD), normalized/cross-correlation (NCC/CC), normalized/mutual information (NMI/MI), or other divergence-based or information-theoretic measures [3]. However, it is not sufficient to rely only on similarity metric, because the solution does not ensure any spatial correlation between the adjacent points. Such high dimensional transformations involved in nonrigid registration make the problem ill-posed. Therefore, additional regularizing constraints are required to enable a reasonable estimation of the displacement field. In general, given the target image *B* and the template image *A*, the common form of deformable registration problem is

(1) $$\begin{array}{c} T^{*}=\underset{T\in \Gamma }{\arg\;\min}\;E\left(T\right)=\underset{T\in \Gamma }{\arg\;\min}\;E_{\text{sim}}\left(B,A\circ T\right)+E_{\text{reg}}\left(T\right). \end{array}$$

The first term *E*_sim_(*B*, *A*∘**T**) in (1) measures the similarity between the deformed template image and the target image. The second term *E*_reg_(**T**), the regularization constraint, ensures the minimization problem is to be well-posed. The set Γ is the space of admissible transformations. The optimal transformation T* ∈ Γ is obtained by minimizing the overall cost function, where Γ is the space of admissible transformations.

The regularization constraint plays crucial role in nonrigid registration problem. Different constraints have been proposed in the literature. References [4, 5] introduced inverse consistency as regularization constraint, which can be explained that the composition of the optimal forward transformation and the backward transformation between the template image and the target image is the identity. Smooth deformation field is also a commonly used constraint [6, 7] against noise. Reference [8] used an incompressibility constraint ensured by limiting the Jacobian determinant of a transformation should be unity. Diffeomorphic transformation is studied much in recent years [9–11], that means continuous, differentiable, and reversible transformation. If the transformation is diffeomorphic, the deformed image should be topologically preserved. Miller et al. [12] proposed the group of diffeomorphic mappings for fluid flow registration. The optimal solution is obtained by regularizing the velocity field to avoid singular solution in numerical implementation. LDDMM [13] aims at finding smooth diffeomorphic mapping for large deformation by searching the smallest geodesic distance.

Direct topology-preserving constraint is another important regularization for nonlinear image registration. The main intuition behind topology preservation in a deformation field is the desire to maintain connectivity between neighboring morphological structures. One way to ensure the topology unchanged during deformation is to keep the Jacobian determinant of the transformation always positive. The other way is to identify a credible deformation space according to the registration model and then to find the optimal transformation in the credible deformation space. Reference [14] derived elegant linear constraints that provide necessary and sufficient conditions to ensure that the Jacobian determinant values of such transformations are positive everywhere. Reference [15] extended the work of [14] to 3D B-splines deformations. They adopted an interval analysis to find the maximum reasonable step along optimal searching path to ensure positive Jacobian determinant. Reference [16] considered enforcing topology preservation as a hard constraint at several intermediate steps of a deformable registration procedure or after the registration was done. Reference [17] used a large-scale constrained optimization method to solve the registration problem. By adding conditions involving the gradient of the Jacobian determinant, this method encouraged the topology preserving to be achieved everywhere.

Over the past years, more and more studies have investigated the nonrigid image registration problem with proper regularization energies. Nevertheless, the existing research is far from being mature. There are still different drawbacks. Some methods need to track the discrete Jacobian determinant or its gradient, which significantly increases computational cost. Some deformable registration models are built in continuous domain. Although topology preservation holds for the continuous transformation, it is no longer guaranteed when the practical solution is obtained on the discrete image grid. Here, we propose a new regularization scheme for nonrigid image registration to hold topology preservation. The details are organized as follows.  Section 2 illustrates the entire scheme of the proposed nonrigid registration algorithm.  Section 3 provides the experimental results and evaluations of the registration algorithm. The conclusion is drawn in Section 4.

## 2. Methods

**Figure 1:**
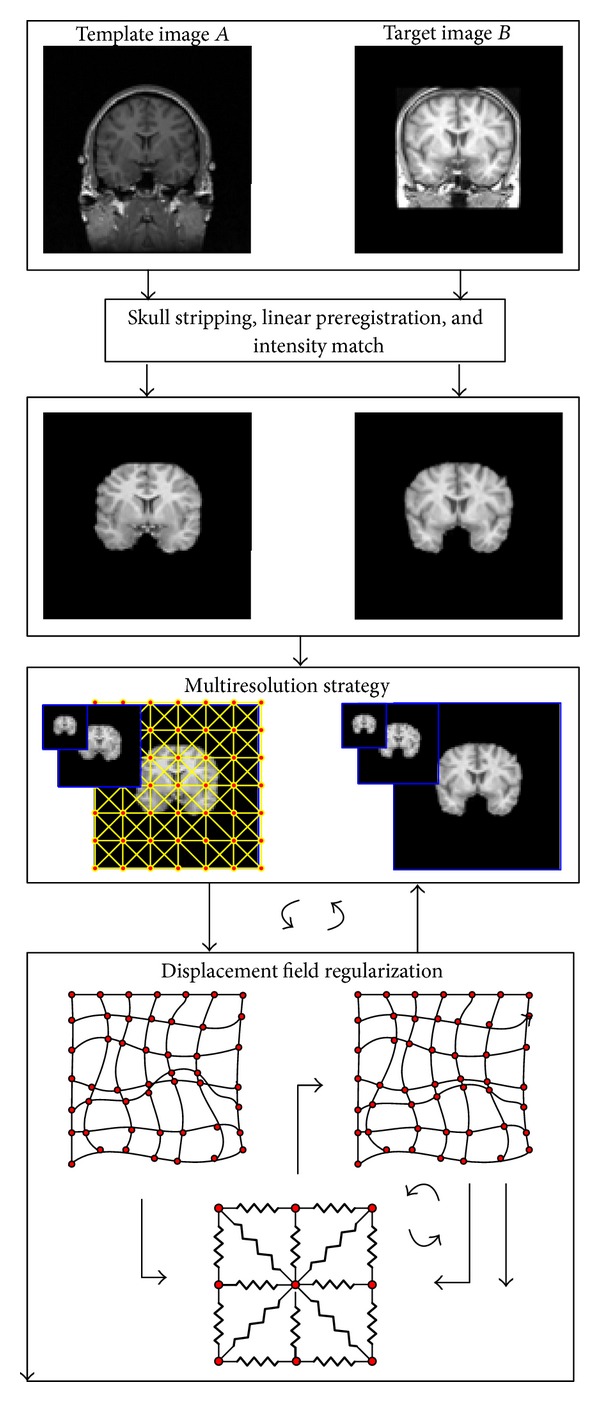
The framework of the NR-MDC algorithm.

The nonrigid image registration model proposed here focuses on maintaining the unchanged topology of the deformed image. A desirable property of intersubject medical image warping is the preservation of the topology of anatomical structures. From medical perspective, some normal homology tissue or structure for any individual, such as internal brain structures, should have the same topology. A topology-preserving transformation guarantees the unchanged connectivity inside a structure and the relationships between the neighboring structures in the deformed image. There is no tearing, no folding, and no appearance or disappearance of structures. To achieve such performance, we propose to cover the deformable template image with a triangular mesh. The triangular mesh is generated according to the position rather than the value of control points. Then the topological relations are controlled by limiting the mesh deformation. The advantage is that the algorithm will get a better deformation quality even if no explicit Jacobian determinant constraint is used. The entire procedure of NR-MDC is showed in Figure 1. The registration procedure employs iterative style: extraction rough displacement field by measurements of similarity without considering spatial relations between points and optimization the displacement field by measurements of regularization energy. A multiresolution strategy propagating solutions from coarser to finer scales is used here to speed up the convergence of the algorithm and to avoid local optima.

### 2.1. Similarity Measures

Different features can be used to establish a similarity metric between the deformed template image and the target image to guide the deformed image towards the target image [3]. Here we use SSD as the similarity metric because it is simple and easy to deal with. The SSD forms the basis of the intensity-based image registration algorithms, and the optimal solution can be obtained by classical optimization algorithms [18]. It can be written as

(2) $$\begin{array}{c} E_{\text{sim}}\left(B,A\circ T\right)=\sum_{p\in \Omega }{\left(B\left(p\right)-A\circ T\left(p\right)\right)}^{2}, \end{array}$$

where **p** is pixel position, **p** = (*x*, *y*), and *Ω* is the image domain. Optical flow field theory [19] is usually adopted to find the displacement field. To better understand the method, we will describe its basic principle roughly. Suppose an object evolving over time, and *I*(**p**(*t*), *t*) is the images of this object. Based on intensity conservation assumption, the image function satisfies

(3) $$\begin{array}{c} I\left(p\left(t\right),t\right)=\text{const}. \end{array}$$

By differentiating (3) with respect to *t*,

(4) $$\begin{array}{c} \frac{\partial I}{\partial x}\frac{\partial x}{\partial t}+\frac{\partial I}{\partial y}\frac{\partial y}{\partial t}=-\frac{\partial I}{\partial t}. \end{array}$$

Consider image *A*∘**T** and *B* being two samples of *I*(**p**(*t*), *t*) and let the sampling time to be unit time, then

(5) $$\begin{array}{c} v\left(p\right)\cdot \nabla B\left(p\right)=A\circ T\left(p\right)-B\left(p\right), \end{array}$$

where **v**(**p**) = (∂*x*/∂*t*, ∂*y*/∂*t*) is the object moving velocity. By approximation, the velocity can be expressed as

(6) $$\begin{array}{c} v\left(p\right)=\frac{\left(A\circ T\left(p\right)-B\left(p\right)\right)\nabla B\left(p\right)}{{||\nabla B\left(p\right)||}^{2}}. \end{array}$$

Generally, the displacement of the point **p** is **u**(**p**) = −**v**(**p**). To avoid unstable solution for small values of ∇*B*(**p**), (6) can be renormalized to

(7) $$\begin{array}{c} v\left(p\right)=\frac{\left(A\circ T\left(p\right)-B\left(p\right)\right)\nabla B\left(p\right)}{{||\nabla B\left(p\right)||}^{2}+{\left(A\circ T\left(p\right)-B\left(p\right)\right)}^{2}}. \end{array}$$

SSD metric is based on the implicit assumption that the intensities of two corresponding points in images *A* and *B* are equal. However this condition is seldom fulfilled in real-world medical image registration, because there are many factors that may affect observed intensity of a tissue over the imaged field, such as the different scanner or scanning parameters, normal aging, different subjects, and so on. In view of such situation, intensity normalization is necessary. In addition, small motion assumption should be satisfied in optical flow field theory. To reduce the difference between the template image and the target image as much as possible, it is better to do spatial normalization using rigid or affine transformation before using (7) to complete the registration.

### 2.2. Regularization

The deformation field obtained from similarity measures does not consider any spatial relations among neighboring points. The points' interdependencies should be guaranteed by certain regularization constraints. Here we focus on topology preservation constraints. It is one basic topology property that the connection relationship between points and edges is unchanged in topology transformation. As is analyzed in [20], three main behaviors, fold, cross, and tear, will cause topological changes during deformation. To eliminate the unfavorable defects, an extra topology correction procedure was done in [20] by tracking the Jacobian determinant of the deformation field. Thus computational cost will increase inevitably.

The basic motivation of our work is to establish a topology preservation transformation in discrete domain directly and to avoid calculating the Jacobian determinant from time to time. To do that, we assume to cover the deformable image with a suitable mesh. The central idea of the proposed method is to deform the template image in terms of the similarity metric, while controlling the deformation range according to the intrinsic topologies of the triangular mesh. That means for each pixel in image level there is a corresponding mesh node to control its motion. The crucial problem is to control mesh deformation perfectly.

Research about mesh deformation was done in computational fluid dynamics [21, 22]. The common applications in engineering include deformable aircraft, airfoil pneumatic elastic vibration, bionic flow, and so on. To satisfy the topology preservation transformation requirements, we adopt segment spring analogy dynamic mesh technology [23], which is initially used to deform a mesh around a pitching airfoil. Its basic idea is to replace each mesh edge by a spring. Thus the vertex motion will be controlled by all the springs connected to this vertex.

Suppose the mesh edge between two adjacent vertices, *i* and *j*, to be a line spring. Initially, the mesh is in static equilibrium state with the spring equilibrium length equals to the edge length. Given an external force along the spring, the spring length will change. According to Hook's Law, the force at vertex *i* will be

(8) $$\begin{array}{c} F_{ij}=k_{ij}\left(u_{j}-u_{i}\right), \end{array}$$

where *k*_*ij*_ is the spring stiffness between *i* and *j* and **u**_*j*_ is the displacement of vertex *j*. Influenced by all the neighboring vertices, the composition of forces at vertex *i* is

(9) $$\begin{array}{c} F_{i}=\sum_{j\in n_{v}}F_{ij}=\sum_{j\in n_{v}}k_{ij}\left(u_{j}-u_{i}\right), \end{array}$$

where *n*_*v*_ is the vertex set whose element connects to vertex *i* directly. For the mesh to be in equilibrium state again, the force at each vertex should be zero. That is

(10) $$\begin{array}{c} \sum_{j\in n_{v}}k_{ij}\left(u_{j}-u_{i}\right)=0. \end{array}$$

Regrouping (10) yields

(11) $$\begin{array}{c} u_{i}=\frac{1}{\sum_{j\in n_{v}}^{}k_{ij}}\sum_{j\in n_{v}}k_{ij}u_{j}. \end{array}$$

In an iterative style, (11) can be rewritten as

(12) $$\begin{array}{c} u_{i}^{n+1}=\frac{1}{\sum_{j\in n_{v}}^{}k_{ij}}\sum_{j\in n_{v}}k_{ij}u_{j}^{n}, \end{array}$$

where *n* denotes the iterative number. Then the new position of vertex *i* is

(13) $$\begin{array}{c} x_{i}^{n+1}=x_{i}^{n}+u_{i}^{n+1}. \end{array}$$

**Figure 2:**
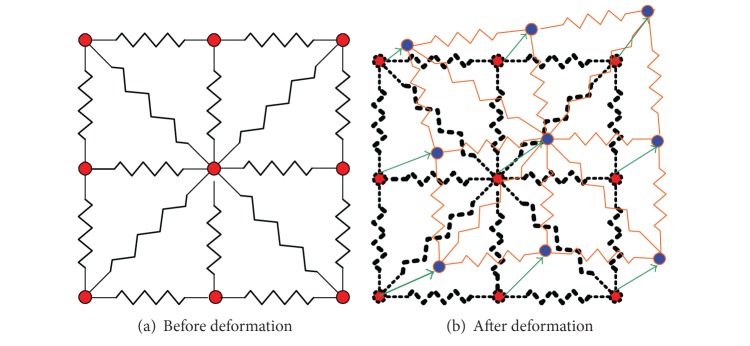
The interpretation of the spring analogy.

The interpretation of the segment spring analogy deformation behavior is showed in Figure 2. The green arrow represents the vertex displacement vector from previous position (red dot) to next position (blue dot).

As can be seen from (12), the displacement vector of vertex *i* is calculated as a weighted result of the neighboring vertices' displacement. The weighting value is determined by the spring stiffness. There are different ways to choose the spring stiffness. For simplicity, the spring stiffness proposed by Batina [23] is used here, which is inversely proportional to the edge length. Consider,

(14) $$\begin{array}{c} k_{ij}=\frac{1}{\left|x_{j}-x_{i}\right|}. \end{array}$$

As is analyzed in [24], this spring stiffness diminishes the probability of vertex collision during the mesh deformation. This means that the factors causing topological changes during deformation (fold, cross, and tear) will also be reduced.

Introducing segment spring analogy into nonrigid medical image registration as a regularization constraint, the expression can be written as

(15) $$\begin{array}{c} E_{\text{reg}}=\sum_{p\in \Omega }\left|\sum_{j\in n_{v}}k_{ij}\left(u_{j}-u_{i}\left(p\right)\right)\right|, \end{array}$$

where index *i* represents vertex *i*, whose position is **p**.

Therefore the problem is described as

(16) T∗=arg min⁡T∈Γ⁡∑p∈Ω(B(p)−A∘T(p))2+∑p∈Ω|∑j∈nvkij(uj−ui(p))|,

(17) $$\begin{array}{c} T=x+u. \end{array}$$

### 2.3. Implementation

The registration energy function (16) is the sum of two measures. The first term is the sum of scalar, while the second term is the sum of vector. One can minimize this function with respect to **u** simultaneously. However, it is not a trivial work. Fortunately, alternating minimization can be used to approximate the optimal solutions [7, 25]. First, the rough displacement field **u** is found by minimizing the similarity metric term. Then the optimized displacement field $\tilde{u}$ is found by minimizing the regularization term. This strategy enables the partial minimizations quite fast. In addition, to avoid falling into local minima and to reduce the computational cost, multiresolution framework is adopted. The image is downsampled into several different scales. The registration starts from the coarsest scale to the finest scale. Accordingly, the resulting deformation transfers from the coarsest scale to the finest scale by upsampling. Here the resampling factor is set to be 2, and the image dimensions of the coarsest scale should not be too small. The primary algorithm implementation procedure is summarized as follows.

*Algorithm Implementation*

1. Given the template image *A* and the target image *B*, perform preprocess procedure, which includes
  - skull stripping using the BrainSuite software [26];
  - linear spatial normalization using FSL software [27];
  - intensity normalization using histogram match.
2. Decompose the images into different scales. For each position at the coarsest scale, set the initial displacement field **u** ^*n*^ = 0, *n* = 0:
  - compute the transient displacement field **u** using (7);
  - get the rough displacement field **u** ^*n*+1^ = **u** ^*n*^ + **u**;
  - get the regularized displacement field ${\tilde{u}}^{n+1}$ using (12);
  - repeat step (a)–(c) until convergence;
  - transfer to the next finer scale, and repeat step (a)–(d) until the finest scale is processed.
3. If there exists a negative Jacobian determinant value of the final deformation field, do 3–5 iterations using (12) to optimize the displacement field.
4. If the similarity between the deformed template image and the target image does not reach the established criteria, update the template image using the obtained transformation and repeat steps (2)–(3).

## 3. Results

The proposed nonrigid registration approach is evaluated on real individual's MRI images and compared with Diffeomorphic Demons algorithm (DD) [28]. The reference image used in the experiment is the one offered by the Surgical Planning Laboratory of Harvard Medical School [29]. It consists of 256 × 256 × 160 voxels with a spatial resolution of 0.9375 mm × 0.9375 mm × 1.5 mm. The test images are real brain MRI images of fifteen normal subjects provided by the Center for Morphometric Analysis at Massachusetts General Hospital and available from Internet Brain Segmentation Repository (IBSR) [30].

### 3.1. Evaluation of the Topology Preservation Ability

During the registration, both a criterion based on the cross-correlation (noted as CC_*t*_) and the iteration number constitute the iteration stopping criteria. If the cross-correlation between the deformed reference image and the target image equals or exceeds CC_*t*_ or the iteration number of reregistration using the deformed image as the new reference image reaches the upper limit (here it is three, while within a scale, the iteration number is set to be ten), the algorithm will stop. CC_*t*_ is defined as

(18) $$\begin{array}{c} \text{C}{\text{C}}_{t}=\frac{\left(1-\text{C}{\text{C}}_{0}\right)}{\alpha }+\text{C}{\text{C}}_{0}, \end{array}$$

where CC_0_ is the initial cross-correlation between the reference image and the target image. The value of 1.2 is suitable for parameter *α* in the experiment.

**Figure 3:**
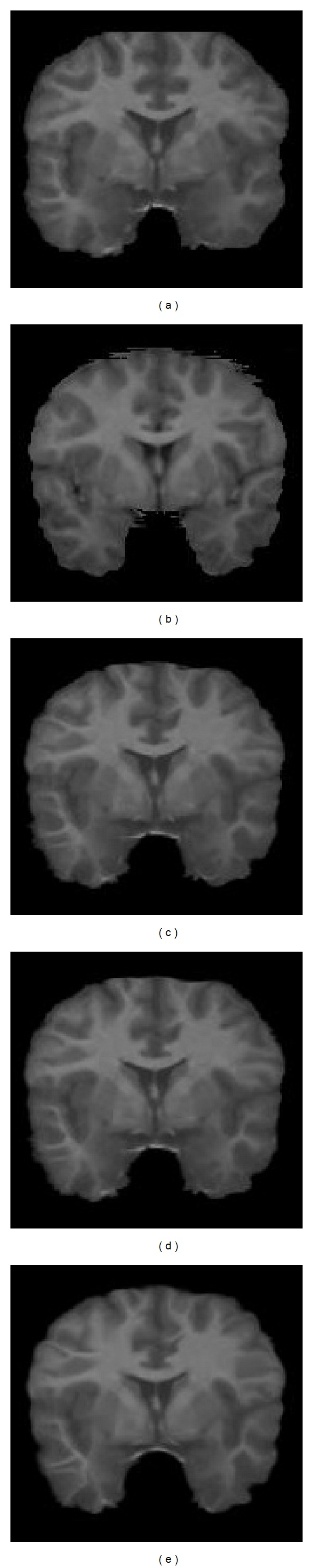
Brain MRI images registration results using NR-MDC algorithm. (a) Template image, (b) target image, (c) result without additional regularization, (d) result without updating the template image, and (e) result with 3 times updating the template image.

**Figure 4:**
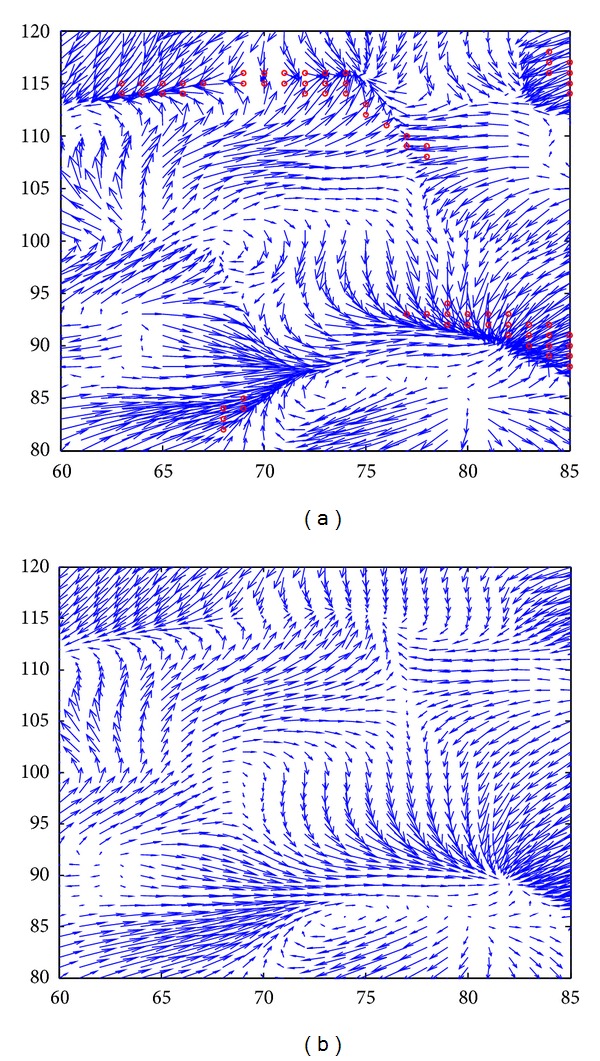
The deformation field using NR-MDC algorithm (a) without additional regularization and (b) with additional regularization.

Figures 3 and 4 show some typical registration results.  Figure 3 gives a visual inspection of the registration result using NR-MDC algorithm. Obviously, the deformed template image with updating scheme (Figure 3(e)) is more similar to the target image (Figure 3(b)) than the other two results (Figures 3(c) and 3(d)).  Figure 4 compares the local deformation field before and after regularization, where the red circles (Figure 4(a)) mark out the locations with negative Jacobian determinant. It can be seen that after several iterations, the regularized deformation field becomes realistic.

Table 1 compares the NR-MDC algorithm, Diffeomorphic Demons algorithm and method in [20]. In Table 1, the criterion CC (cross-correlation factor) depicts the similarity degree between the deformed template image and the target image. CC = 1 means the maximum similarity. The criterion *N*_*J*_ depicts the number of points with a negative Jacobian determinant of the deformation field when compared to the total point. S1 represents the registration result without additional regularization. S2 represents the registration result without updating the template image. S3 represents the registration result with regularization and updating steps. Since the vertex motion has high-freedom degree and each vertex is closely related to its surrounding vertices, immediate deformation regularization is inadequate. There still exists a small amount of points with changed topologies. Thus in most cases, additional regularization is necessary. Fortunately, less iteration is required to achieve acceptable deformations. In addition, the need for template image updating and the updating number depend on both the initial difference between the template image and the target image after global linear registration and the expected similarity. Proper updating will improve the similarity. The DD algorithm is carried out in a multiresolution manner. The main parameters are three scales; regularization with a Gaussian convolution kernel, whose standard deviation is set to be one; the maximum template image updating number is three. It can be seen from Table 1 that the average CC value is 0.984 for NR-MDC algorithm using S3 strategy, and the deformation field is topologically preserved. While for the DD algorithm, the average CC value is about 0.97, and there are still a small amount of points with negative Jacobian determinants. Since an additional Jacobian determinant tracking procedure was carried out in the method of [20], the algorithm running time is relatively long.

### 3.2. Evaluation of the Brain Internal Structures Segmentation Ability

To further evaluate the reasonability of the obtained deformation field, the brain internal structures segmentation experiment is carried out. The segmented structures are left and right caudate (L-Caudate, R-Caudate), putamen (L-Putamen, R-Putamen), thalamus (L-Thalamus, R-Thalamus), and hippocampus (L-Hippocampus, R-Hippocampus). As is known, these brain subcortical structures have relatively small sizes, complex shapes. Moreover, there is only small spacing between different structures, while their intensities in MRI images are very similar. All these negative factors make the fully automatically accurate segmentation a challenging task.

To validate the results quantitatively, a kappa statistic-based similarity index, Dice coefficient, is adopted in this paper. The similarity index measures the overlap ratio between the segmented structure and the ground truth, which is defined as

(19) $$\begin{array}{c} \text{KI}=\frac{2\times \text{TP}}{2\times \text{TP}+\text{FN}+\text{FP}}. \end{array}$$

The definitions of the parameters are as follows:

 TP = *G*∩*E*: the number of true positive;
 
$\text{FP}=\overset{-}{G}\cap E$: the number of false positive;
 
$\text{FN}=G\cap \overset{-}{E}$: the number of false negative;

where *G* is the ground truth segmentation of a given structure, *E* is the estimated segmentation of the same structure, and $\overset{-}{O}$ denotes the complement of a set *O*. Perfect spatial correspondence between the two segmentations will result in KI = 1, whereas no correspondence will result in KI = 0.

The results are presented in Table 2, where the KI values are the mean values of all the volumes. The results indicate that the proposed NR-MDC algorithm gives better segmentations than the DD algorithm.

**Figure 5:**
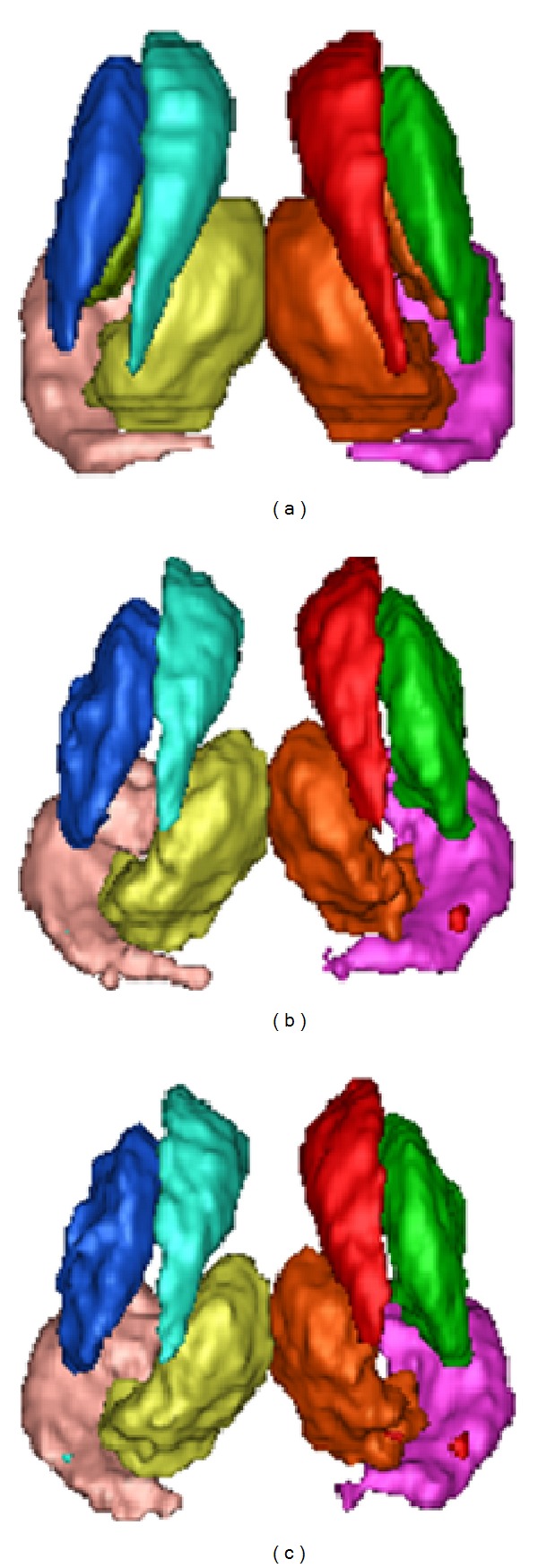
Typical 3D views for the segmented structures: (a) “ground truth” segmentation, (b) NR-MDC segmentation, and (c) DD segmentation.

Typical 3D views for the segmented structures are presented in Figure 5.

## 4. Conclusions

In this paper, a new nonrigid medical image registration method, named NR-MDC, is proposed. A new deformable source, a triangular mesh, is added to cover the template image. This mesh is independent of the image intensities. It reflects the points' intrinsic spatial relations and is used to regularize the basic deformation field computed from the image intensity information. The proposed cost function is optimized in an alternative minimization way. The deformation field is first computed by optimizing a SSD metric and then is regularized by using spring analogy method. This approach enforces a valid topology of the deformed mesh, which means a valid image deformation. To evaluate the performance of NR-MDC algorithm, intersubject brain MRI image registration experiments are done. And a comparative experiment is also done between the proposed method and the state-of-the-art DD algorithm. The results verify NR-MDC method's excellent deformation ability, as well as its good topology-preserving ability.

## Figures and Tables

**Table 1:** Comparison of the topology preservation ability between NR-MDC algorithm and DD algorithm.

Algorithm	Strategy	CC	*N*_*J*_	Time (minutes)
NR-MDC	S1	0.971	0.5%	14.8
	S2	0.966	0%	15.3
	S3	0.984	0%	41.7
DD		0.972	0.03%	54.1
[20]		0.979	0%	84.6

**Table 2:** Comparison of averaged KI value between our NR-MDC algorithm and DD algorithm.

	L-Caudate	R-Caudate	L-Putamen	R-Putamen	L-Thalamus	R-Thalamus	L-Hippocampus	R-Hippocampus
NR-MDC	0.728	0.778	0.749	0.755	0.746	0.779	0.729	0.691
DD	0.710	0.762	0.732	0.741	0.722	0.754	0.711	0.682
